# Clinical evaluation of performance, biocompatibility, and safety of vitamin E-bonded polysulfone membrane hemodialyzer compared to non-vitamin E-bonded hemodialyzer

**DOI:** 10.1007/s10047-019-01110-w

**Published:** 2019-06-26

**Authors:** Mercedeh Kiaii, Masaharu Aritomi, Mitsuyo Nagase, Myriam Farah, Beverly Jung

**Affiliations:** 1 St. Paul’S Hospital, 1081 Burrard Street, Vancouver, BC Canada; 2 Asahi Kasei Medical Co., Ltd., 1-1-2 Yurakucho, Chiyoda-ku, Tokyo, 100-0006 Japan

**Keywords:** Hemodialysis, Intradialytic hypotension, Platelet, Good clinical practice, Adverse event

## Abstract

The vitamin E-bonded polysulfone membrane hemodialyzer (ViE™-21) was evaluated in a clinical study for regulatory submission. Seventeen patients on hemodialysis were treated with conventional high-flux hemodialyzers for 2 weeks (Pre-ViE phase) and switched to the ViE-21 for 36 sessions (ViE phase) followed by an additional 2 weeks on conventional hemodialyzers (Post-ViE phase). Reduction ratios of urea, creatinine, beta-2-microglobulin, albumin, and ultrafiltration coefficients (KUF) were measured once during the Pre-ViE phase and twice during the ViE phase. Moreover, biocompatibility markers [leucocyte count, platelet count, and activated complement factor (C3a) levels] were evaluated pre-dialysis, 15 min after initiation, and post-dialysis. During the study, type and number of adverse events (AEs), and device malfunctions were recorded. ViE-21 reduction ratios and KUF were not noticeably different than those of conventional hemodialyzers. Fluctuations of leucocyte counts and C3a concentrations were similar using ViE-21 and conventional hemodialyzers; however, the platelet count fluctuation was lower in ViE-21 sessions. The frequency of episodes of hypotension occurring during the ViE phase was lower than that occurring during the Pre- and Post-ViE phases. In conclusion, this study provided performance and safety data of the ViE-21 for regulatory application. The data suggest that vitamin E-bonded hemodialyzers are beneficial in lowering platelet activation and frequency of intradialytic hypotension. Larger randomized controlled trials are needed to confirm these findings.

## Introduction

Patients suffering from end-stage renal disease (ESRD) require renal replacement therapy to remove uremic toxins from their blood. Hemodialysis, an extra-corporeal blood purification therapy using a hemodialyzer, is a well-accepted treatment for ESRD patients. In the early stages, hollow fibers of cellulose were used as membrane materials for hemodialyzers. However, when blood components and cells come in to contact with the cellulose membrane, it causes significant activation of complement and reduction of leucocyte counts from the peripheral blood during the dialysis treatment. To overcome such bio-incompatibility, synthetic polymer membranes such as a polysulfone or polyether sulfone membrane were developed [1, 2].

Another challenge of hemodialysis is to reduce oxidative stress during the extracorporeal treatments. Anemia, cardiovascular disease, chronic inflammation, and intradialytic hypotension are among the common complications in patients receiving hemodialysis, which show a causal relationship with oxidative stress [3]. To reduce oxidative stress, vitamin C and E are well-accepted supplements having antioxidant activities.

Consequently, polysulfone-based vitamin E-bonded membranes were developed [4-8] to achieve a synergistic effect of the biocompatibility of synthetic membranes and the antioxidant activity of vitamin E. This clinical study was designed to evaluate the vitamin E-bonded polysulfone hemodialyzer, ViE™-21, manufactured by Asahi Kasei Medical Co., Ltd., at a clinical site in Canada to obtain performance and safety data for regulatory submission.

## Methods

### Design of the study

The study titled “Clinical Study of Asahi ViE Dialyzer in Canada (AVID)” was conducted as a prospective, open-label, non-randomized, single-arm study to satisfy regulatory requirements for single-use hemodialyzer in accordance with the Food and Drug Administration (FDA) guidance [9] of the United States of America, and in compliance with Good Clinical Practice of International Organization for Standardization (ISO) 14155:2011 guidelines [10]. The study was approved by the research ethics board of the University of British Columbia–Providence Health Care Research Institute, Vancouver, British Columbia, Canada, and registered at ClinicalTrials.gov with the registration number NCT 02292212. For each patient, data were collected over six dialysis sessions on a conventional hemodialyzer, which was the same hemodialyzer used for routine dialysis therapy (Pre-ViE phase), and then patients were switched to ViE-21 (surface area: 2.1 m^2^) for 36 dialysis sessions (ViE phase) for further data collection. After completion of the ViE phase, patients were switched to the original hemodialyzer used during the Pre-ViE phase for a final 6 dialysis sessions (Post-ViE phase) to observe safety issue which might be originated from ViE-21 treatments.

### Patients

Up to 20 patients were to be enrolled at the start of the study to ensure that at least 12 patients completed 36 treatments each on the ViE-21 to satisfy regulatory requirements [9]. The inclusion criteria were: age between 18 and 80 years; stable condition on maintenance hemodialysis for at least 12 weeks; expectation to remain on hemodialysis for at least 24 weeks; on hemodialysis for more than 3 h per treatment and on a 3 times-per week schedule; having vascular access either as a native arteriovenous fistula or graft; well-maintained and capable of obtaining blood flow rate equal to or more than 350 mL/min; use of high-flux dialyzers (KUF ≥ 40 mL/h/mmHg) with surface area ≥ 1.5 m^2^ and ≤ 2.2 m^2^; capable of understanding the informed consent form; and provision of written consent and willingness to participate in the study. Blood flow rate was specified to meet criteria of the FDA guidance [9]. Exclusion criteria included: medical conditions requiring regular blood transfusion; history of more than 1-week hospitalization related to infection; inflammation or surgery within the past 12 weeks; previous participation in another clinical investigation within the previous 12 weeks; difficulty in maintaining vascular access function within the previous 12 weeks; known to be hepatitis B, C, or human immunodeficiency virus-positive; female patients who were pregnant, breast feeding, or planning a pregnancy within the study period; received blood purification therapy other than conventional hemodialysis within the past 12 weeks; intolerant to heparin; and any serious medical, social or psychological condition that in the opinion of the investigator would disqualify a patient from participation.

### Reduction ratio evaluation

Blood samples were taken to assess reduction ratios of urea, creatinine, beta-2-microglobulin (B2M), and albumin once in the Pre-ViE phase during the 2nd (Wednesday or Thursday) or 3rd (Friday or Saturday) session of the 1st week for the conventional hemodialyzer, and twice in the ViE phase during weeks 7 and 13 at the 2nd or 3rd session for the ViE-21. The basis of selecting 2nd or 3rd session of the weeks was to unify interdialytica period before sampling. Pre-dialysis blood samples were collected within 15 min prior to commencing dialysis and post-dialysis blood samples were collected within 15 min after the completion of dialysis. The blood samples were immediately centrifuged to separate plasma, and then the plasma was used to assay the concentrations of urea, creatinine, B2M, and albumin. Reduction ratios of urea and creatinine were calculated using the following formula:

$${\text{Reduction ratio }}\left( \% \right) \, = \, \left[ {\left( {C_{{{\text{pre}}}} - C_{{{\text{post}}}} } \right)/ \, \left( {C_{{{\text{pre}}}} } \right)} \right] \times 100$$

where, *C*_pre_ and *C*_post_ were the urea or creatinine concentrations in peripheral blood at pre- and post-dialysis phases, respectively. The reduction ratios of B2M and albumin were calculated using the following formula with hematocrit (HCT) correction.

$${\text{Reduction ratio }}\left( \% \right) \, = \, \left\{ {1 \, - \, \frac{\left[ {{\text{HCT}}_{{{\text{pre}}}} \times \left( {1 \, - {\text{ HCT}}_{{{\text{post}}}} \, / \, 100} \right) \times C_{post} } \right]}{\left[ {{\text{HCT}}_{{{\text{post}}}} \times \left( {1 \, - {\text{ HCT}}_{{{\text{pre}}}} \, / \, 100} \right) \times C_{pre} } \right]}} \right\} \times 100$$

where, *C*_pre_ and *C*_post_ were the concentrations of B2M and albumin in peripheral plasma, and HCTpre and HCTpost were HCT values (in %) measured at pre- and post-dialysis sessions, respectively.

For urea reduction evaluation, the values of Kt/V were determined using the following formula:

$$\begin{aligned} \text{Kt}/\text{V} \, &= \, - {\ln}\left[ {\left( {C_{post} /C_{pre} } \right) - 0.008 \times t }\right]\\ &\quad { \, + \, } \left[ 4 - 3.5 \times \left( {C_{{{\text{post}}}} /C_{{{\text{pre}}}} } \right) \right] \times \left( {{\text{dBW}}/{\text{BW}}} \right) \end{aligned}$$

where, *t* was dialysis treatment time (in hours), BW was body weight (dry weight in kg), and dBW was the difference in body weight (in kg) between pre- and post-dialysis.

### KUF evaluation

The KUF values were measured once in the Pre-ViE phase at the 1st or 2nd week for the conventional hemodialyzer, twice during the ViE phase at weeks 3–8, and at weeks 9–14 for the ViE-21. Each evaluation was performed at either the 2nd or 3rd treatment session but not on the same day of blood sampling for reduction ratio evaluation. The measurements were performed in accordance with the FDA guidance [9] as the recording of transmembrane pressure values 10 min after adjustment of ultrafiltration rates to 600, 1000, 1400, and 1800 mL/h in 10 min intervals, respectively. KUF values were then computed by measuring the slope of the ultrafiltration rate versus the transmembrane pressure for each patient using least squares estimates.

### Biocompatibility evaluation

Biocompatibility was evaluated by measuring blood leucocyte and platelet counts as well as C3a concentrations in plasma. Blood samples were taken pre-dialysis, 15 min after initiation, and post-dialysis. Blood samples were collected once in the Pre-ViE phase at the 2nd or 3rd session of the 1st week for the conventional hemodialyzer, twice in the ViE phase during weeks 7 and 13, and at the 2nd or 3rd treatment sessions for the ViE-21 during the same day of blood sample collection for reduction ratio evaluation. Blood samples collected for C3a determinations were centrifuged to separate plasma, and then the separated plasma was stored frozen until all samples from all patients in the study were collected. The samples for C3a testing were all analyzed at one time to minimize test variability.

### AE and device malfunction

All AEs were recorded for all treatments and interdialytic days in a precise manner. For example, minor events such as a patient’s feeling of discomfort or muscle cramps requiring minimal intervention, such as changing ultrafiltration flow rate, were recorded as an AE in the case report forms. The dialysis treatment condition parameters such as flow rates of blood and dialysate, vital signs at pre- and post-dialysis, dry weight, volume of water removed, arterial and venous pressure of the extracorporeal circuit, use of anticoagulant therapy, and concomitant medications were recorded. All device malfunctions occurring during any of the treatment sessions were also recorded.

### Statistical analysis

In the study protocol, statistical analysis was not foreseen, however, for the post hoc analysis the following statistical analyses were conducted. The two sets of $\text{Kt}/\text{V}$, KUF, as well as reduction ratios of urea, creatinine, B2M, and albumin data at the ViE phase were compared to the corresponding data at the Pre-ViE phase using paired *t* test, respectively. For the biocompatibility markers, after normalization by setting the pre-dialysis levels as baseline (100%) and after HCT correction, the two sets of ViE phase data of leucocyte, platelet, and C3a levels were compared to those of the Pre-ViE phase data using the paired *t* test. No adjustment for multiple comparisons was performed due to the post hoc nature of the analysis. The *P* values were indicated in the results section without judging statistical significance. The statistical analyses were performed using SAS version 9.4 (SAS Institute Inc., USA).

## Results

### Patient enrollment and analysis population

A total of 74 patients were screened for enrollment. Fifty-one patients were determined not to be eligible and an additional 6 patients declined to participate. Therefore, the study population consisted of 17 patients. Of the 17 patients enrolled, all completed the baseline assessments and Pre-ViE phase. Three patients began the ViE phase but exited the study prior to completing this phase. One patient was withdrawn after the 14th session because the patient was incorrectly given hemodiafiltration, while this study was restricted to hemodialysis only. A second patient was withdrawn because the patient accidently received 4 consecutive dialysis treatments using the conventional hemodialyzer instead of the ViE-21 during the ViE phase. The third patient withdrew consent at the 29th session. Therefore, 14 patients participated in all 3 phases of the study as per the established protocol. None of the patients were lost to follow-up and no deaths occurred during the study. In the following evaluations, the data for all 17 patients were defined as the safety analysis (SAA) population and the data for the 14 patients who participated in all 3 study phases were defined as the intent-to-treat (ITT) population. The first treatment of the study began on January 5, 2015 and the last treatment was completed on November 6, 2015.

### Performance evaluation

The reduction ratios of urea, creatinine, B2M, and albumin as well as Kt/V and KUF values for the ITT population including *P* values of paired *t* tests comparing the single Pre-ViE phase measurement with two ViE phase measurements are summarized in Table 1. As indicated in Table 1, there were no detectable differences among the ITT values with the exception of three values: the reduction ratio of urea for the second ViE phase was lower, the reduction ratio of albumin for the first ViE phase was lower, and the KUF value for the first ViE phase had a higher tendency compared to the values for the Pre-ViE phase with *P* values of 0.048, 0.038, and 0.028, respectively. These three tendencies were not observed for the other ITT values in ViE phase with *P* values of 0.395, 0.110, and 0.103, respectively. The differences of type and surface area of the conventional hemodialyzers at the Pre-ViE phase were FX 600 (*n* = 1), FX 800 (*n* = 7), FX 1000 (*n* = 5) (Fresenius Medical Care, polysulfone membrane, surface areas were 1.5, 1.8, and 2.2 m^2^, respectively), and Nephral ST500 (*n* = 1) (Baxter, polyacrylonitrile membrane, surface area was 2.15 m^2^). The mean and standard deviation of dry weights of the patients were 63.5, 66.5 ± 9.5, 93.5 ± 15.5, and 68.0 kg for the groups of FX 600, FX 800, FX 1000, and ST500, respectively. The ST500 was used for patients who had demonstrated sensitivity to the polysulfone membrane. The differences of hemodialyzers used at the Pre-ViE phase did not show noticeable differences in the values of reduction ratios, as well as Kt/V and KUF as shown in Table 1.

**Table 1:** Reduction ratios, Kt/V, and ultrafiltration coefficient (KUF) values

Phase	Pre-ViE phase		ViE phase 1st measurement			ViE phase 2nd measurement		
Dialyzer	Conventional		ViE-21			ViE-21		
Reduction ratio (%)	Mean	± SD	Mean	± SD	*P*^a^	Mean	± SD	*P*^a^
Urea^b^								
ITT^c^ (*n* = 14)	76.1	± 5.4	74.4	± 8.8	0.395	73.5	± 4.5	0.048
FX 600^d^ (*n* = 1)	81.4	–	79.2	–		78.9	–	
FX 800^d^ (*n* = 7)	77.6	± 4.2	77.4	± 10.4		74.7	± 3.8	
FX 1000^d^ (*n* = 5)	73.9	± 6.6	69.9	± 6.7		70.4	± 4.8	
Nephral ST500^d^ (*n* = 1)	70.9	–	71.7	–		74.5	–	
Creatinine^b^								
ITT^c^ (*n* = 14)	70.7	± 4.6	70.0	± 8.4	0.686	69.0	± 5.4	0.192
FX 600^d^ (*n* = 1)	75.7	–	74.2	–		74.5	–	
FX 800^d^ (*n* = 7)	72.4	± 4.1	73.5	± 9.4		71.2	± 4.3	
FX 1000^d^ (*n* = 5)	68.3	± 4.4	64.8	± 6.0		64.6	± 5.0	
Nephral ST500^d^ (*n* = 1)	65.2	–	67.1	–		69.9	–	
Beta-2-microglobulin^e^								
ITT^c^ (*n* = 14)	65.5	± 6.6	65.8	± 8.8	0.906	65.9	± 6.5	0.841
FX 600^d^ (*n* = 1)	70.6	–	68.5	–		72.3	–	
FX 800^d^ (*n* = 7)	68.2	± 1.8	70.5	± 7.9		67.1	± 4.1	
FX 1000^d^ (*n* = 5)	63.2	± 8.9	58.7	± 7.4		61.9	± 8.6	
Nephral ST500^d^ (*n* = 1)	54.0	–	65.4	–		71.2	–	
Albumin^e^								
ITT^c^ (*n* = 14)	2.4	± 6.4	− 2.4	± 3.9	0.038	− 1.0	± 4.4	0.110
FX 600^d^ (*n* = 1)	2.1	–	0.3	–		− 2.5	–	
FX 800^d^ (*n* = 7)	0.3	± 3.9	− 1.9	± 3.8		− 0.3	± 3.9	
FX 1000^d^ (*n* = 5)	4.9	± 9.7	− 2.1	± 3.1		− 0.4	± 5.4	
Nephral ST500^d^ (*n* = 1)	4.0	–	− 10.5	–		− 6.9	-	
Kt/V^f^								
ITT^c^ (*n* = 14)	1.70	± 0.28	1.79	± 0.95	0.705	1.56	± 0.19	0.062
FX 600^d^ (*n* = 1)	1.99	–	1.86	–		1.84	–	
FX 800^d^ (*n* = 7)	1.76	± 0.26	2.09	± 1.29		1.59	± 0.17	
FX 1000^d^ (*n* = 5)	1.60	± 0.31	1.43	± 0.25		1.45	± 0.18	
Nephral ST500^d^ (*n* = 1)	1.46	–	1.44	–		1.66	–	
KUF (mL/h/mmHg)								
ITT^c^ (*n* = 14)	61.2	± 13.6	67.8	± 8.6	0.028	68.6	± 9.3	0.103
FX 600^d^ (*n* = 1)	76.9	–	65.2	–		61.4	-	
FX 800^d^ (*n* = 7)	60.7	± 6.2	68.3	± 5.0		63.5	± 7.7	
FX 1000^d^ (*n* = 5)	64.8	± 15.1	70.8	± 11.0		75.6	± 7.2	
Nephral ST500^d^ (*n* = 1)	30.3	–	51.2	–		77.1	–	

^a^*P* values indicate the results of paired *t* test comparing ITT values of ViE and Pre-ViE phases

^b^Reduction ratios of urea and creatinine are indicated without hematocrit correction

^c^ITT: intent to treat

^d^Each data of ITT population were divided in the groups categorized by the type of conventional dialyzers in Pre- and Post-ViE phases

^e^Reduction ratios of beta-2-microglobulin and albumin are indicated with hematocrit correction

^f^No unit of quantity required for $\text{Kt}/\text{V}$

### Biocompatibility evaluation

**Fig. 1:**
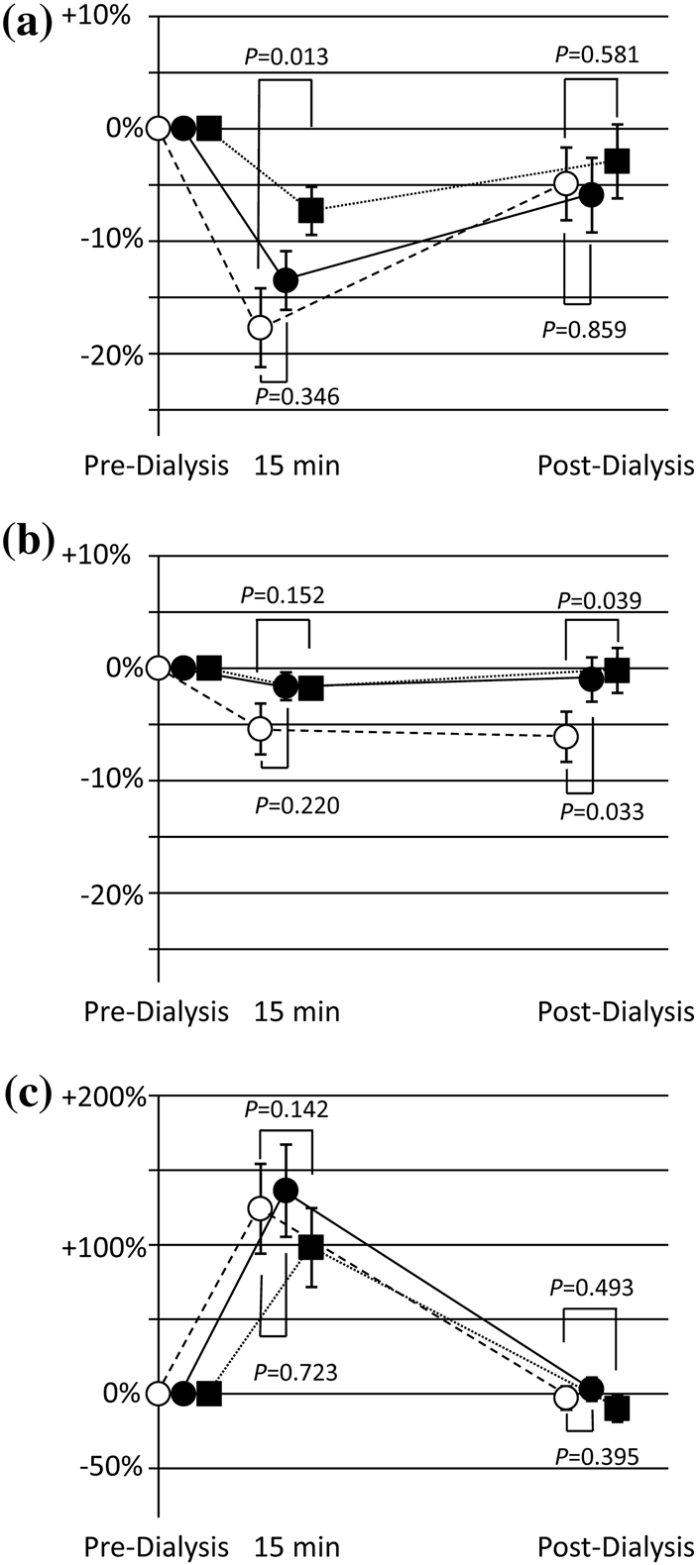
Fluctuation of biocompatibility markers during the dialysis sessions for the intent-to-treat (ITT) population. Figures **a**,** b**, and **c** correspond to fluctuations of leucocyte, platelet, and C3a, respectively. Open circles, filled circles, and filled squares indicate measurements at the Pre-ViE phase, ViE phase 1st measurement, and ViE phase 2nd measurement, respectively. The 15 min after initiation and post-dialysis values were first normalized by defining the pre-dialysis values as baseline (100%) and using the hematocrit correction equation. The changes (%) at 15 min and post-dialysis are shown in the figure by indicating the pre-dialysis point as 0%. The bars indicate standard errors and *P* values indicate the results of paired *t* test compared to the Pre-ViE phase

Fluctuations of leucocyte, platelet, and C3a levels during dialysis session are illustrated in Fig. 1. Both the Pre-ViE and ViE phase measurements showed a reduction of leucocyte levels at 15 min after the initiation of dialysis treatment averaging 10–20% and a return to the pre-dialysis level at post-dialysis. Regarding the platelet levels, the Pre-ViE phase measurement showed an approximately 5% reduction at both 15 min after initiation and at post-dialysis, while both ViE phase measurements showed smaller fluctuations. Both post-dialysis levels of the ViE phase measurements almost returned to pre-dialysis levels, while post-dialysis levels of the Pre-ViE phase measurements did not recover to pre-dialysis levels, and showed a greater than 5% reduction. For complement activation evaluated by C3a levels, all Pre-ViE and ViE phase measurements showed activation of about 100–150% at 15 min after initiation of treatments with the return to the pre-dialysis levels at post-dialysis. The values of these biomarkers showed no tendency of deterioration nor improvement throughout the study period as indicated in Table 2.

**Table 2:** Biocompatibility markers during dialysis session for the intent-to-treat (ITT) population

Phase	Pre-ViE phase		ViE phase 1st measurement		ViE phase 2nd measurement	
Dialyzer	Conventional		ViE-21		ViE-21	
	Mean	± SD	Mean	± SD	Mean	± SD
Leucocyte (10^9^ cells/L)						
Pre-dialysis	7.2	± 2.1	7.1	± 1.4	7.2	± 1.8
15 min after initiation	6.0	± 2.0	6.2	± 1.6	6.7	± 1.9
Post-dialysis	6.9	± 2.0	6.6	± 1.3	6.9	± 1.6
Platelet (10^9^ cells/L)						
Pre-dialysis	200.7	± 46.6	205.7	± 62.3	205.3	± 57.3
15 min after initiation	189.7	± 47.5	201.4	± 56.9	200.4	± 51.1
Post-dialysis	187.5	± 42.9	200.7	± 51.5	202.7	± 46.9
C3a (ng/mL)						
Pre-dialysis	78.8	± 24.1	70.7	± 23.3	101.2	± 111.1
15 min after initiation	160.6	± 60.2	153.2	± 54.6	141.1	± 48.5
Post-dialysis	74.3	± 27.5	70.8	± 25.3	68.0	± 20.7

Values are indicated as mean ± standard deviation (SD) based on the intent-to-treat (ITT) population (*n* = 14). Each values of leucocyte counts, platelet counts, and activated complement factor (C3a) concentrations at 15 min after initiation of dialysis or post-dialysis sessions were corrected by hematocrit values (HCT) using the following equations:

WBC_cor_ = WB_Cori_ × (HCT_pre_/HCT_time_)

PLT_cor_ = PLT_ori_ × (HCT_pre_/HCT_time_)

C3a_cor_ = C3a_ori_ × (HCT_pre_/HCT_time_)

where, HCT_pre_ is HCT at pre-dialysis and HCT_time_ are HCT at 15 min after initiation or post-dialysis. Besides, WBC_cor_, PLT_cor_, and C3a_cor_ are corrected values and WBC_ori_, PLT_ori_, and C3a_ori_ are original values of leucocyte count, platelet count, and C3a concentration, respectively

### AE evaluation

The summary of the AEs for the SAA population is shown in Table 3. In all phases of the study, AEs were commonly observed with proportions varying between 78.6 and 100% of patients having at least one event. Most of the AEs were related to the dialysis procedures themselves and the remainder were related to other non-dialysis-related factors. Two of the AEs were related to the ViE-21 and occurred in a single patient. The patient began the study having 7 dialysis treatments with the conventional hemodialyzer, then switched to the ViE-21 for treatment session 8 and continued with the ViE-21 through treatment session 28. During treatment session 26, the patient experienced a mild event of pruritus. The pruritus progressed and during the next treatment session 27, the patient experienced a full-body rash. The patient was given 25 mg of diphenhydramine during the dialysis session. The next day, the patient was given another 50 mg of diphenhydramine. The patient also switched back to the conventional hemodialyzer for session 29 and then withdrew from the study by patient’s decision. The rash did not resolve after the diphenhydramine administration and the patient started taking prednisone 2 days after treatment session 29 for 10 days. The event was considered resolved after about 3 weeks from session 26. There were no other identified changes to the patient’s treatment to explain the body rash, and it is possible that this was a delayed hypersensitivity reaction to ViE-21. Among the AEs, one event was categorized in SAE. The patient complained of weakness, dizziness, and nausea during dialysis at session 47 in the Post-ViE phase. Additional descriptions of the symptoms included vertigo and generalized body weakness. The patient was treated with dimenhydrinate and discharged. This was coded as vertigo and was judged to be unrelated to the ViE-21 or the conventional hemodialyzer, but with an unknown relationship to the dialysis procedure.

**Table 3:** Summary of adverse events (AEs)

	Sessions with AEs						Patients with AEs					
	Pre-ViE phase		ViE phase		Post-ViE phase		Pre-ViE phase		ViE phase		Post-ViE phase	
	Session	(%)^a^	Session	(%)^a^	Session	(%)^a^	Patient	(%)^b^	Patient	(%)^b^	Patient	(%) ^b^
AEs	55	53.9	216	39.4	42	50.0	16	94.1	17	100.0	11	78.6
ViE-21-related	0	0.0	2	0.4	0	0.0	0	0.0	1	5.9	0	0.0
Conventional hemodialyzer-related	0	0.0	0	0.0	0	0.0	0	0.0	0	0.0	0	0.0
Dialysis procedure-related	43	42.2	158	28.8	31	36.9	15	88.2	17	100.0	9	64.3
Other related events	12	11.8	58	10.6	11	13.1	8	47.1	15	88.2	6	42.9
Total number of sessions or patients	102		548		84		17		17		14	

All AE counts observed in the safety analysis (SAA) population (*n* = 17) were evaluated

^a^Percent (%) means the number of sessions with AEs divided by total number of sessions

^b^Percent (%) means the number of patients with AEs divided by total number of patients

### Occurrence proportions of AEs

The occurrence proportions of all or each symptom during the study for the SAA population are summarized in Table 4. The frequency of dialysis sessions with any AE was lower in the ViE phase (29.7%) compared to Pre- and Post-ViE phases (36.6%). Specifically, the frequency of sessions with hypotension, which was the most common AE in all phases, was lower in the ViE phase at 17.0% compared to that in the Pre- and Post-ViE phases at 24.7%.

**Table 4:** Session counts and frequency of adverse events (AEs)

	Pre- and Post-ViE phases (Total session number = 186)			ViE phase (Total session number = 548)		
	No AE^a^	With AE^a^	Frequency (%)	No AE^a^	With AE^a^	Frequency (%)
All AE	118	68	36.6	385	163	29.7
AE symptom^b^						
Hypotension	140	46	24.7	455	93	17.0
Muscle spasms	172	14	7.5	522	26	4.7
Fluid overload	177	9	4.8	520	28	5.1
Dizziness	178	8	4.3	535	13	2.4
Dyspnoea	183	3	1.6	545	3	0.5
Hypertension	184	2	1.1	545	3	0.5
Vomiting	185	1	0.5	545	3	0.5
Nausea	186	0	0.0	545	3	0.5
Back pain	186	0	0.0	544	4	0.7
Pruritus	186	0	0.0	546	2	0.4
Angina pectoris	186	0	0.0	546	2	0.4
Dyspepsia	186	0	0.0	546	2	0.4
Pain	186	0	0.0	546	2	0.4

^a^ “No AE” was defined as sessions without adverse event occurrence in the safety analysis (SAA) population (*n* = 17), and “With AE” was defined as the number of sessions with adverse event occurrence

^b^AE symptoms were coded according to the medical dictionary for regulatory activities (MedDRA), ver. 16.1. The cases of only one session with an AE symptom during the study period were omitted

### Device malfunctions

There were three device malfunctions recorded in one patient in the ViE phase. These three device malfunctions were due to thrombus formation. These events occurred more than 3 h after the initiation of dialysis in a single patient with long dialysis sessions. The sessions were continued after replacement of the ViE-21 and completed without any further problems.

### Anticoagulant-free sessions

During the study, there were three sessions performed without anti-coagulant administration. All these sessions occurred during the ViE phase using ViE-21 for three different patients. The decision not to use anticoagulation was made by the clinician to reduce the risk of bleeding due to recent falls and potential head trauma. All three sessions were completed with no problems, particularly, without thrombus formation or system clotting.

## Discussion

Based on this study, the ViE-21 can be used as a high-flux hemodialyzer with essentially comparable performance to conventional high-flux hemodialyzers. Allergic type reactions were observed in the ViE phase in one patient; however, the symptoms were not severe. Such allergic-type reactions are commonly observed in these complex hemodialysis patients and can be observed with all hemodialyzer types. Although three episodes of dialyzer clotting were observed in one patient during the ViE-phase, retrospective chart review showed that this patient also had problems with dialyzer clotting when conventional hemodialyzers were used before the study. Furthermore, no serious AEs related to the ViE-21 were recorded. Therefore, ViE-21 are equally safe compared to conventional polysulfone high-flux hemodialyzers.

Regarding biocompatibility, ViE-21 showed no detectable differences compared to conventional hemodialyzers on leucocyte and C3a fluctuations during the dialysis treatments. However, ViE-21 showed more stability in platelet fluctuations compared to conventional hemodialyzers, and this may indicate a lower activation property of platelets. Previous studies have demonstrated that vitamin E-bonded polysulfone hemodialyzers were successful at reducing anticoagulation requirements in dialysis treatments [11, 12] and showed comparable anticoagulation with heparin-coated hemodialyzers [13]. An in vitro study reported that vitamin E-bonded membranes might reduce platelet activation driven by leucocytes [14]. During this study, three anticoagulation-free sessions were successfully performed using ViE-21. Therefore, vitamin E-bonding on polysulfone membranes may reduce platelet activation during dialysis sessions and may reduce the need for anticoagulant use during dialysis therapy.

Another interesting finding was that the occurrence of AEs during the ViE phase was lower than during Pre- and Post-ViE phases. In particular, the frequency of hypotensive events was reduced by about 30% in the ViE-phase compared to the Pre- and Post-ViE phases. In previous reports, symptoms of intradialytic hypotension were improved using vitamin E-bonded hemodialyzers [15, 16]; and thus, these hemodialyzers may potentially reduce the incidence of hypotension.

In this study, there were no findings showing a reduction of the dosage requirement for erythropoiesis stimulating agents (ESA) or improvement of hemoglobin level by post hoc analysis, even though previous studies demonstrated such improvement in ESA resistance [17-24]. This may be due to the observation period consisting of 3 months, which was not sufficient to optimally evaluate improvements in anemia. Also, previous studies of vitamin E-bonded polysulfone hemodialyzers have demonstrated a reduction in oxidative and inflammatory markers [18, 25-27]; however, this was not evaluated in the present study.

## Conclusion

This study provides performance and safety data for the ViE-21. Vitamin E-bonded polysulfone membrane hemodialyzers may have a beneficial role in reducing platelet activation and lowering the frequency of AEs such as hypotension during hemodialysis treatment. Limitations of this study include the small sample size, and the variations in the surface area of the hemodialyzers used. To confirm these findings, randomized controlled trials with proper comparison design are required.
